# Emerging Therapies Targeting Lipoprotein(a): A Clinical Trial Landscape Review of Investigational Lp(a)-Lowering Therapies

**DOI:** 10.3390/jcm15135233

**Published:** 2026-07-04

**Authors:** Reema M. Alotaibi, Rimas H. Al-Salmi, Renad O. Shosho, Yahya A. Alzahrani, Maan H. Harbi

**Affiliations:** 1 College of Pharmacy, Umm Al-Qura University, Makkah 21955, Saudi Arabia; s443000556@uqu.edu.sa (R.M.A.); s443006547@uqu.edu.sa (R.H.A.-S.); s443002308@uqu.edu.sa (R.O.S.); 2 Department of Pharmacology, Faculty of Medicine, King Abdulaziz University, Rabigh 25732, Saudi Arabia; yaaalzahrani1@kau.edu.sa; 3 Department of Pharmacology and Toxicology, College of Pharmacy, Umm Al-Qura University, Makkah 21955, Saudi Arabia

**Keywords:** Lipoprotein(a), Lp(a), pelacarsen, olpasiran, lepodisiran, zerlasiran, muvalaplin, antisense oligonucleotides, small interfering RNA, cardiovascular disease, clinical trials

## Abstract

**Background/Objectives**: Elevated lipoprotein(a) [Lp(a)] is an independent cardiovascular risk factor associated with atherosclerotic cardiovascular disease and calcific aortic valve disease. Historically, therapeutic options for reducing Lp(a) have been limited. This study aimed to characterize the clinical development landscape of emerging Lp(a)-targeted therapies, evaluate endpoint assessment strategies, and summarize available efficacy evidence from investigational agents. **Methods**: A qualitative clinical trial landscape review was conducted using ClinicalTrials.gov. Interventional Phase I–III studies evaluating therapies specifically targeting Lp(a) were identified through a structured registry search performed on 5 November 2025. Eligible studies were screened according to predefined inclusion and exclusion criteria. Extracted data included trial characteristics, therapeutic class, endpoint methodologies, and published efficacy outcomes. Data were synthesized narratively. **Results**: Twenty clinical trials met the eligibility criteria. Three therapeutic classes were identified: antisense oligonucleotides (ASOs), small interfering RNA (siRNA)-based therapies, and small-molecule inhibitors. Pelacarsen represented the sole ASO program, whereas siRNA-based therapies constituted the largest therapeutic category. Five studies were designed as cardiovascular outcomes trials. Percent change from baseline in circulating Lp(a) concentration was the most frequently used efficacy endpoint. Published data demonstrated substantial reductions in Lp(a) concentrations across all major therapeutic platforms. Available non-head-to-head published evidence showed substantial Lp(a) reductions across several investigational agents, including siRNA-based therapies, pelacarsen, and muvalaplin, although differences between studies preclude direct comparison between therapeutic platforms. **Conclusions**: The Lp(a) therapeutic landscape has rapidly evolved, with RNA-based therapies demonstrating unprecedented reductions in circulating Lp(a) concentrations. Ongoing cardiovascular outcomes trials will determine whether these reductions translate into meaningful cardiovascular benefits, establish Lp(a) as a therapeutic target in cardiovascular prevention and clarify the long-term safety and risk–benefit profile of Lp(a)-targeted therapies.

## 1. Introduction

Cardiovascular disease (CVD) remains the leading cause of morbidity and mortality worldwide despite substantial advances in prevention, diagnosis, and treatment [1]. Although traditional risk factors such as hypertension, diabetes mellitus, smoking, and elevated low-density lipoprotein cholesterol (LDL-C) account for a significant proportion of cardiovascular risk, considerable residual risk persists even among patients receiving guideline-directed therapy [2]. In recent years, lipoprotein(a) [Lp(a)] has emerged as an important independent cardiovascular risk factor associated with atherosclerotic cardiovascular disease (ASCVD), myocardial infarction, ischemic stroke, peripheral arterial disease, and calcific aortic valve stenosis. Elevated Lp(a) concentrations are largely genetically determined and remain relatively stable throughout life, distinguishing Lp(a) from many conventional lipid biomarkers that are more responsive to lifestyle modification and pharmacological intervention [2,3].

Lp(a) is a low-density lipoprotein-like particle consisting of an apolipoprotein B-100-containing lipoprotein covalently linked to apolipoprotein(a) [apo(a)] [3,4]. The unique structural characteristics of apo(a), including its homology with plasminogen, contribute to the proatherogenic, proinflammatory, and potentially prothrombotic properties of Lp(a) [3,4]. Epidemiological, genetic, and Mendelian randomization studies have consistently demonstrated a causal relationship between elevated Lp(a) levels and cardiovascular disease [3,5,6]. Furthermore, elevated Lp(a) has been recognized as a major contributor to residual cardiovascular risk, particularly among individuals who have achieved recommended LDL-C targets yet continue to experience cardiovascular events [2,3,4].

Despite increasing recognition of its clinical significance, therapeutic options for reducing Lp(a) have historically been limited [4]. Conventional lipid-lowering therapies such as statins have little effect on Lp(a) concentrations and may even modestly increase levels in some patients [3,5]. While PCSK9 inhibitors can reduce Lp(a) by approximately 20–30%, these reductions are generally insufficient for patients with markedly elevated concentrations [7,8]. Lipoprotein apheresis remains an option for selected high-risk individuals but is resource-intensive, costly, and not widely available [9,10]. Consequently, the absence of effective, targeted therapies has represented a major unmet need in cardiovascular medicine.

Recent advances in molecular therapeutics have transformed the Lp(a) treatment landscape [4]. Novel therapies specifically designed to reduce hepatic production of apolipoprotein(a) or interfere with Lp(a) particle formation have demonstrated unprecedented reductions in circulating Lp(a) concentrations [11,12,13]. These therapies include antisense oligonucleotides (ASOs), small interfering RNA (siRNA)-based agents, and emerging small-molecule inhibitors. Among the most advanced agents currently in clinical development are pelacarsen, olpasiran, lepodisiran, zerlasiran, and muvalaplin, several of which have demonstrated reductions in Lp(a) exceeding 80–90% in clinical studies [11,13,14]. Importantly, multiple large-scale cardiovascular outcomes trials are now underway to determine whether these substantial biomarker reductions translate into meaningful reductions in cardiovascular events [12,15,16].

Several recent reviews have comprehensively discussed Lp(a) biology, cardiovascular risk, and emerging Lp(a)-lowering therapies while also highlighting key ongoing clinical development programs [17,18,19,20]. However, these publications have primarily adopted mechanistic, therapeutic, or trial-design perspectives rather than providing a structured registry-based synthesis of the active phase I–III Lp(a)-targeted clinical development pipeline. The present review complements prior work by using ClinicalTrials.gov as a primary data source to characterize registered interventional studies, compare trial-level design features and endpoint assessment strategies, and summarize the current landscape of cardiovascular outcomes programs and published efficacy evidence across major investigational Lp(a)-lowering platforms. Accordingly, this review aimed to provide a structured assessment of the contemporary Lp(a)-targeted therapeutic pipeline and identify key trends in clinical development, endpoint methodology, and emerging efficacy evidence.

## 2. Materials and Methods

### 2.1. Study Design and Data Source

A qualitative clinical trial landscape review was conducted using the ClinicalTrials.gov registry to identify investigational therapies targeting lipoprotein(a) [Lp(a)]. ClinicalTrials.gov was selected as the registry source because it is a large publicly accessible clinical trial registry that captures many interventional studies evaluating emerging Lp(a)-lowering therapies. Accordingly, this review should be interpreted as a ClinicalTrials.gov-based landscape review rather than a comprehensive global registry review.

The review aimed to characterize the current development pipeline of Lp(a)-lowering therapies, including trial characteristics, endpoint assessment strategies, and available efficacy evidence from published clinical studies. Because the primary objective was to characterize the clinical development landscape rather than quantitatively synthesize treatment effects, a formal risk-of-bias assessment was not performed.

The ClinicalTrials.gov database was searched on 5 November 2025. To enhance reproducibility, the ClinicalTrials.gov search was performed using the Advanced Search interface with the following fields and filters: Condition/Disease field: (“cardiovascular disease” OR “atherosclerosis” OR “hyperlipidemia”); Other Terms field: (“Lipoprotein(a)” OR “Lipoprotein a” OR “Lp(a)” OR “Lp a” OR “Lipoprotein little a” OR “Apolipoprotein(a)” OR “Apolipoprotein a” OR “Apo(a)” OR “Apo a” OR “LPA gene”); Study Type: Interventional; Phase: Phase 1, Phase 1/Phase 2, Phase 2, Phase 2/Phase 3, or Phase 3. No restrictions were applied for country, sex, age group, sponsor, or recruitment status at the initial search stage. Withdrawn, suspended, and terminated studies were excluded during eligibility screening rather than during the initial search.

### 2.2. Eligibility Criteria

Eligible studies were interventional clinical trials evaluating therapies specifically designed to reduce Lp(a) concentrations or investigate the clinical consequences of Lp(a) lowering. Studies were required to include Lp(a) reduction as a primary, secondary, or key exploratory outcome measure. Trials investigating antisense oligonucleotides, small interfering RNA (siRNA) therapies, small-molecule inhibitors, or other targeted Lp(a)-lowering approaches were eligible for inclusion.

Studies were excluded if they were observational studies, non-interventional registries, genetic association studies without a therapeutic intervention, or trials in which Lp(a) was measured only incidentally without relevance to the intervention mechanism or study objectives. Trials with withdrawn, suspended, or terminated status were excluded. Duplicate records, extension studies without distinct safety or efficacy objectives, and studies lacking sufficient registry information to determine intervention type, phase, or Lp(a)-related outcomes were also excluded.

### 2.3. Screening and Study Selection

Investigators (R.M.A., R.H.A., and R.O.S.) independently screened ClinicalTrials.gov records according to the predefined inclusion and exclusion criteria. Eligible studies were identified based on study design, therapeutic target, intervention characteristics, and relevance to Lp(a)-lowering therapy. Following the initial screening process, all included and excluded records were independently reviewed by the principal investigator (M.H.H.) to verify eligibility decisions and ensure consistency in study selection. Discrepancies were resolved through discussion and consensus among the investigators. When necessary, a senior reviewer (Y.A.A.) adjudicated unresolved disagreements. The final study set was approved before data extraction.

### 2.4. Data Extraction

Data extraction was initially performed by the investigators (R.M.A., R.H.A., and R.O.S.) using a standardized extraction template developed for this review. Extracted variables included trial identifier, investigational agent, therapeutic class, study phase, recruitment status, enrollment size, target population, endpoint characteristics, and cardiovascular outcomes trial (CVOT) status. Cardiovascular outcomes trials (CVOTs) were defined as studies primarily designed to evaluate major adverse cardiovascular events (MACEs) or other clinical cardiovascular outcomes rather than pharmacodynamic Lp(a)-lowering endpoints. All extracted data were subsequently verified by the principal investigator for accuracy and completeness. Any discrepancies were resolved through discussion and consensus prior to final data synthesis. Additional treatment-related characteristics, including route of administration, dose levels, dosing frequency, masking, and allocation, were extracted and are presented in Supplementary Table S1.

Published efficacy evidence was identified from the linked “Publications,” “Results Posted,” and reference sections of the corresponding ClinicalTrials.gov records, when available. No separate systematic bibliographic database search was performed. Publications were matched to eligible registered trials using the NCT identifier, investigational agent name, trial acronym, study population, intervention details, and reported Lp(a)-related outcomes. Only peer-reviewed publications corresponding to eligible ClinicalTrials.gov-registered trials were used to summarize published efficacy data.

### 2.5. Data Synthesis

Extracted data were synthesized narratively and organized into three thematic domains. First, trial characteristics were summarized according to therapeutic class, development phase, target population, and study objectives. Second, endpoint assessment methodologies were evaluated to identify common approaches used to quantify Lp(a) reduction and cardiovascular outcomes. Third, published efficacy evidence was reviewed to compare the magnitude, durability, and clinical development status of leading investigational therapies. Results are presented using descriptive summaries and comparative evidence tables.

## 3. Results

### 3.1. Characteristics of Included Clinical Trials

**Figure 1:**
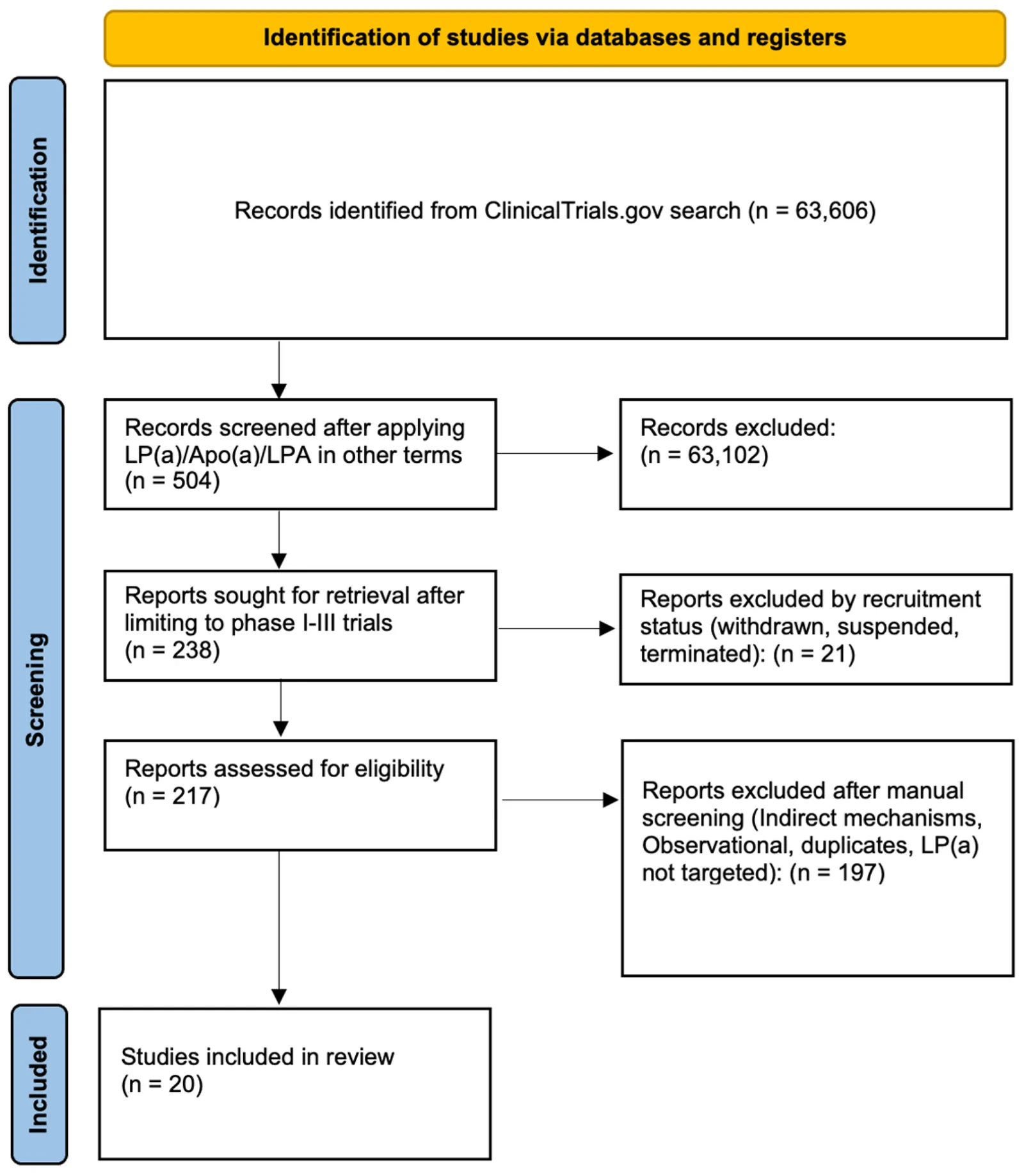
PRISMA flow diagram summarizing the identification and selection of interventional clinical trials of Lp(a)-lowering pharmacological therapies from ClinicalTrials.gov.

A total of 63,606 records were initially identified through ClinicalTrials.gov searches using cardiovascular disease-, atherosclerosis-, and hyperlipidemia-related terms. Application of Lp(a)-specific search terms reduced the dataset to 504 potentially relevant records, of which 238 phase I–III studies were retrieved for further assessment. After exclusion of withdrawn, suspended, and terminated studies (n = 21), 217 records underwent full eligibility assessment. Manual screening excluded 197 records because they evaluated indirect mechanisms, observational designs, duplicate registrations, or interventions not specifically targeting Lp(a). Ultimately, 20 interventional clinical trials investigating targeted lipoprotein(a) [Lp(a)]-lowering therapies met the eligibility criteria and were included in the qualitative analysis (Figure 1).

**Figure 2:**
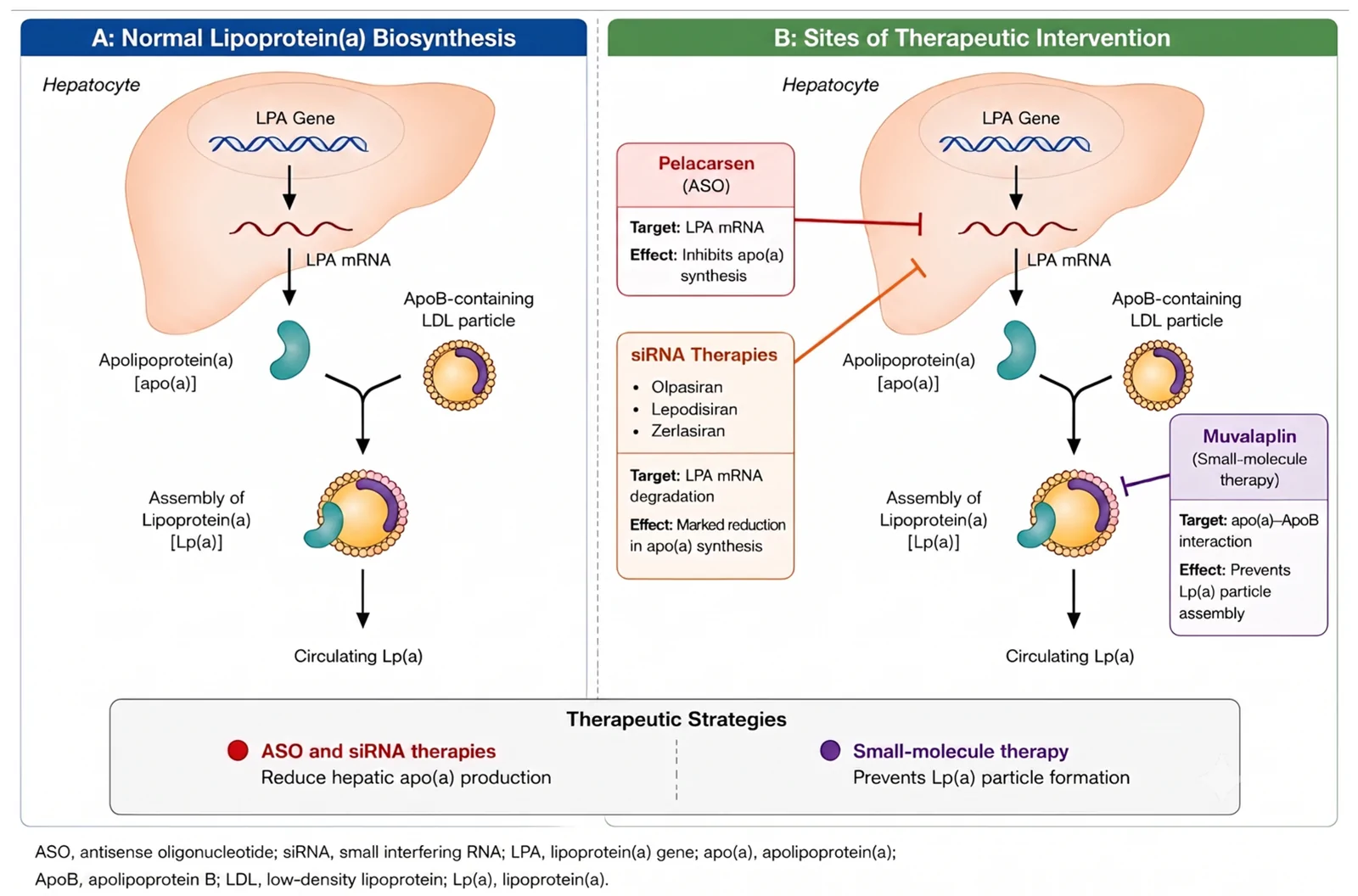
Mechanism of action of therapeutic strategies of emerging lipoprotein(a)-lowering therapies. (**A**) Normal biosynthesis of lipoprotein(a) [Lp(a)], in which hepatocyte-derived apolipoprotein(a) [apo(a)] binds to an apolipoprotein B (ApoB)-containing low-density lipoprotein (LDL) particle to form circulating Lp(a). (**B**) Therapeutic targets of emerging Lp(a)-lowering agents. Pelacarsen (ASO) and siRNA-based therapies (olpasiran, lepodisiran, and zerlasiran) reduce hepatic apo(a) production through inhibition or degradation of *LPA* mRNA, whereas muvalaplin prevents apo(a)-ApoB interaction and inhibits Lp(a) particle assembly.

Included investigational therapies were categorized into three principal mechanistic classes: antisense oligonucleotides (ASOs), small interfering RNA (siRNA) therapies, and oral small-molecule inhibitors. Of the 20 included trials, 8 (40%) evaluated ASO therapies, 9 (45%) evaluated siRNA therapies, and 3 (15%) evaluated small-molecule inhibitors. ASO therapies, primarily represented by pelacarsen, act through targeted inhibition of hepatic LPA mRNA translation, thereby reducing apolipoprotein(a) synthesis [4,13]. siRNA therapies, including olpasiran, lepodisiran, zerlasiran, HRS-5632, and SRSD216, utilize RNA interference mechanisms to degrade LPA messenger RNA and achieve prolonged suppression of lipoprotein(a) production [4,12]. Small-molecule inhibitors, including muvalaplin and HRS-5346, differ mechanistically by disrupting apo(a)-apoB interactions involved in lipoprotein(a) particle assembly [11] (Table 1; Figure 2).

The clinical development landscape spanned Phase 1 through Phase 3 studies. Early-phase investigations primarily focused on safety, tolerability, pharmacokinetics, and pharmacodynamic assessment of Lp(a) lowering, whereas later-phase studies increasingly evaluated long-term efficacy and cardiovascular outcomes. Five studies were classified as cardiovascular outcomes trials based on their primary focus on clinical cardiovascular endpoints rather than pharmacodynamic Lp(a)-lowering outcomes. Sample sizes ranged from 41 participants in long-term extension studies to 16,700 participants in large-scale cardiovascular outcomes trials.

Most studies enrolled participants with elevated Lp(a) concentrations and either established cardiovascular disease or increased cardiovascular risk. Several trials targeted specialized populations, including patients with calcific aortic stenosis, individuals undergoing lipoprotein apheresis, patients with hyperlipoproteinemia(a), and Black/African American and Hispanic populations with elevated cardiovascular risk. Five studies were specifically designed as cardiovascular outcomes trials, including Lp(a) HORIZON, OCEAN(a)-Outcomes, ACCLAIM-Lp(a), MOVE-Lp(a), and a primary prevention outcomes trial evaluating olpasiran [12,15,19].

### 3.2. Lp(a)-Related Endpoint Assessment Strategies

Substantial heterogeneity was observed in endpoint assessment methodologies across the included studies (Table 2). Percent change from baseline in circulating Lp(a) concentration was the most frequently employed efficacy endpoint and was used across multiple ASO, siRNA, and small-molecule development programs. Alternative approaches included log-transformed concentration changes, absolute concentration changes, composite pharmacodynamic assessments, and time-averaged percent reduction analyses.

Among the 20 included trials, percentage change from baseline was the most common endpoint format (6 studies, 30%), followed by composite pharmacodynamic assessments (4 studies, 20%), time-to-event plus Lp(a) reduction endpoints (5 studies, 25%), log-transformed concentration changes (3 studies, 15%), and absolute concentration changes (2 studies, 10%).

Several studies incorporated threshold achievement analyses (5 trials) to determine the proportion of participants attaining predefined Lp(a) targets, while others evaluated broader lipid and biomarker profiles, including apolipoprotein B (ApoB), low-density lipoprotein cholesterol (LDL-C), oxidized phospholipids on apolipoprotein(a) [OxPL-apo(a)], and additional exploratory biomarkers. Safety, tolerability, and pharmacokinetic assessments were common components of early-phase studies (7 trials), reflecting the developmental nature of many investigational agents.

Outcome-driven trials employed a different assessment framework, combining Lp(a) reduction with clinical cardiovascular endpoints. Major adverse cardiovascular events (MACEs) served as the primary clinical outcome measure in several Phase 3 programs, including studies evaluating pelacarsen, olpasiran, lepodisiran, and muvalaplin. Additional disease-specific endpoints were also identified, such as aortic valve progression in calcific aortic stenosis and reduction in lipoprotein apheresis requirements among patients with severe hyperlipoproteinemia(a). Follow-up durations varied substantially, ranging from 12 weeks in early pharmacodynamic studies to more than six years in cardiovascular outcomes trials [15,19].

Administration strategies differed substantially across therapeutic platforms. Pelacarsen was generally administered by monthly subcutaneous injection, several siRNA therapies used subcutaneous dosing schedules compatible with infrequent administration, and small-molecule inhibitors such as HRS-5346 and muvalaplin were administered orally (Table 2). Detailed trial design and treatment characteristics, including allocation, masking, dose levels, dosing frequency, and route of administration, are summarized in Supplementary Table S1.

### 3.3. Comparative Efficacy of Investigational Lp(a)-Lowering Therapies

Published non-head-to-head efficacy data demonstrated substantial reductions in circulating Lp(a) concentrations across several investigational therapeutic platforms (Table 3). The reported maximum reductions included approximately 80% with pelacarsen, approximately 95–100% with olpasiran, approximately 95–97% with lepodisiran, approximately 98–99% with zerlasiran, and approximately 86% with muvalaplin, with sustained suppression maintained for almost 12 months [12,14,21,22]. These values are presented as descriptive summaries of each agent’s reported clinical studies rather than as direct comparative estimates between therapeutic platforms.

Pelacarsen, the most clinically advanced ASO therapy, demonstrated reductions of approximately 80% in Phase 2 studies and remains the furthest-developed agent within the ASO class. Unlike siRNA therapies, pelacarsen requires ongoing administration to maintain Lp(a) suppression, although reductions remained stable throughout chronic treatment. The ongoing Phase 3 Lp(a) HORIZON trial is expected to provide the first definitive evidence regarding whether targeted Lp(a) lowering translates into reductions in cardiovascular events [13].

Muvalaplin represents a mechanistically distinct therapeutic approach and is currently the most advanced oral Lp(a)-lowering agent in clinical development. In Phase 2 studies, muvalaplin achieved reductions of approximately 86% while maintaining the convenience of daily oral administration. The availability of an oral therapeutic option may offer important advantages for long-term treatment adherence and patient acceptance, although its clinical role remains dependent on confirmation of long-term safety and cardiovascular outcome benefit [11].

Collectively, the available evidence indicates that modern Lp(a)-targeting therapies can achieve reductions substantially exceeding those attainable with conventional lipid-lowering therapies. However, despite impressive biomarker reductions, definitive evidence demonstrating cardiovascular outcome benefits remains pending and will depend on the results of ongoing Phase 3 outcomes trials.

## 4. Discussion

This qualitative analysis of ClinicalTrials.gov-registered studies demonstrates the rapid evolution of the Lp(a)-targeted therapeutic pipeline from early pharmacodynamic studies to large-scale cardiovascular outcomes trials. The identified studies encompass antisense oligonucleotides (ASOs), small interfering RNA (siRNA)-based therapies, and small-molecule inhibitors, reflecting growing recognition of Lp(a) as an independent and potentially modifiable cardiovascular risk factor [19,20]. RNA-targeted therapies predominated within the current development landscape and directly target hepatic apolipoprotein(a) production. Available non-head-to-head evidence suggests that several RNA-based therapies achieve profound and durable reductions in Lp(a) concentrations; however, direct comparisons between agents remain inappropriate because studies differed substantially in design, populations, baseline Lp(a) concentrations, dosing regimens, endpoint definitions, assay methodologies, and follow-up duration. The prolonged pharmacodynamic effects reported for some siRNA-based therapies may support infrequent dosing schedules, whereas oral small-molecule approaches may offer practical advantages for patient acceptance and long-term adherence if efficacy and safety are confirmed in later-stage studies [12,14,21].

Pelacarsen remains clinically important because it is the most advanced ASO-based therapy and one of the principal agents being evaluated for cardiovascular event reduction. Its progression into a large outcomes program reflects the maturity of its clinical development and provides an important benchmark for newer Lp(a)-lowering therapies. The results of ongoing outcomes studies will be critical in determining whether targeted pharmacological Lp(a) lowering translates into meaningful reductions in cardiovascular risk [13].

The development of muvalaplin introduces a distinct strategy by targeting Lp(a) particle assembly rather than hepatic apo(a) synthesis. As an orally administered Lp(a)-targeting agent, muvalaplin may broaden future treatment options by offering a non-injectable alternative to RNA-based therapies. However, its clinical role will depend on confirmation of sustained efficacy, long-term safety, assay-based interpretation of treatment response, and cardiovascular outcome benefit in later-stage trials [11].

An additional consideration in the development of Lp(a)-lowering therapies is their safety and tolerability. Available short-term evidence from early-phase clinical studies suggests that ASO-, siRNA-, and small-molecule-based approaches have generally been well tolerated, with no major safety concerns identified to date. Reported adverse events have been predominantly mild to moderate in severity, and treatment discontinuation due to adverse events has been uncommon in published studies. However, these findings should not be interpreted as establishing long-term safety or definitive risk–benefit profiles. Current safety evidence remains limited by relatively small sample sizes, short follow-up durations, and the early developmental stage of several investigational agents. Consequently, the long-term safety implications of sustained and profound Lp(a) suppression remain incompletely understood. Ongoing cardiovascular outcomes trials, including Lp(a) HORIZON, OCEAN(a)-Outcomes, ACCLAIM-Lp(a), and MOVE-Lp(a), will therefore play a critical role not only in determining clinical efficacy but also in establishing the long-term safety, tolerability, and overall risk–benefit profile of these therapies. A summary of currently available safety and tolerability findings from published clinical studies is provided in Supplementary Table S2.

Despite the remarkable reductions in circulating Lp(a) achieved by these investigational agents, an important question remains unanswered: whether substantial biomarker reduction translates into meaningful reductions in cardiovascular events. Although Mendelian randomization, genetic, and epidemiological studies support a causal role of elevated Lp(a) in atherosclerotic cardiovascular disease (ASCVD), the magnitude of cardiovascular benefit achievable through pharmacological Lp(a) lowering remains uncertain [15,19]. The current evidence base is dominated by pharmacodynamic studies demonstrating profound Lp(a) reduction; however, definitive proof of clinical benefit requires large-scale cardiovascular outcomes trials. Several ongoing Phase 3 studies, including Lp(a) HORIZON, OCEAN(a)-Outcomes, ACCLAIM-Lp(a), and MOVE-Lp(a), have therefore become pivotal to the future of the field. The results of these studies will determine whether Lp(a) lowering should become a routine component of cardiovascular risk reduction strategies, clarify the relationship between the magnitude of Lp(a) reduction and cardiovascular benefit, and may influence future guideline recommendations regarding screening and treatment.

Another notable observation is the expanding scope of Lp(a)-targeted research beyond traditional secondary prevention populations. While many studies focus on patients with established cardiovascular disease, several trials are evaluating individuals with elevated Lp(a) in primary prevention settings, patients with calcific aortic stenosis, and individuals requiring lipoprotein apheresis. These investigations reflect growing recognition that the pathological effects of Lp(a) extend beyond atherosclerosis alone [3,9]. In particular, the association between elevated Lp(a) and calcific aortic valve disease has stimulated interest in determining whether targeted Lp(a) reduction may alter disease progression, potentially creating new therapeutic opportunities in valvular heart disease.

Lp(a) measurement remains an important methodological consideration when interpreting Lp(a)-lowering trials. Concentrations may be reported in mass units (mg/dL) or molar units (nmol/L), but direct conversion between these units is unreliable because apo(a) isoforms vary substantially in size due to differences in kringle IV type 2 repeat number. Assay performance may also be influenced by apo(a) isoform heterogeneity, contributing to variability in measured Lp(a) concentrations. Contemporary consensus statements and guideline updates therefore recommend standardized, isoform-insensitive assays whenever possible and encourage reporting in nmol/L to improve comparability across studies and clinical settings [2,23].

These measurement issues have direct implications for cross-trial interpretation. Differences in assay calibration, reporting units, endpoint definitions, and analytical approaches may influence estimated treatment effects and limit direct comparability between studies. Consequently, apparent differences in Lp(a)-lowering efficacy among pelacarsen, olpasiran, lepodisiran, zerlasiran, and muvalaplin should be interpreted cautiously, as observed effect sizes may partly reflect methodological differences rather than true pharmacological superiority. This further reinforces the importance of standardized assessment approaches and ongoing cardiovascular outcomes trials to determine whether Lp(a) reduction translates into clinically meaningful benefit [2,4].

Several important research gaps remain. The optimal magnitude of Lp(a) reduction required for cardiovascular benefit is uncertain, and the long-term safety of maintaining very low Lp(a) concentrations over prolonged periods is not yet established. Future studies should also determine whether genetic or assay-related factors should inform patient selection and treatment monitoring. Comparative effectiveness between therapeutic platforms also remains unknown, including whether differences in potency, durability, dosing frequency, route of administration, cost, accessibility, and adherence will translate into clinically meaningful distinctions. Overall, the available evidence indicates that targeted Lp(a)-lowering therapies have progressed from a theoretical concept to a rapidly advancing clinical reality; however, ongoing outcomes programs will be decisive in determining whether profound biomarker reductions ultimately reduce cardiovascular morbidity and mortality.

### Limitations

This review has several limitations. First, it was restricted to ClinicalTrials.gov and associated published reports; therefore, studies registered exclusively in other sources, including WHO ICTRP, EU CTIS/EudraCT, ISRCTN, or regional registries, may not have been captured. Exclusion of withdrawn, suspended, and terminated studies may also have limited identification of unsuccessful development programs, safety concerns, or operational challenges. Second, because the Lp(a)-targeted therapeutic landscape is rapidly evolving, trial statuses, enrollment figures, protocols, and available efficacy data may have changed after the search date. However, no dedicated cardiovascular outcomes trial of targeted Lp(a) lowering has yet reported definitive primary results, and no Lp(a)-specific therapy has received regulatory approval. Third, several agents remain in early clinical development, and peer-reviewed efficacy data were not available for all included therapies. Published efficacy findings were therefore summarized descriptively and were not adjusted for methodological quality, risk of bias, or certainty of evidence. Finally, substantial heterogeneity in study design, populations, endpoint definitions, assay methodologies, and follow-up duration precluded quantitative synthesis or meta-analysis, and definitive conclusions regarding cardiovascular outcome benefits await completion of ongoing Phase 3 trials.

## 5. Conclusions

The Lp(a)-targeted therapeutic landscape has expanded rapidly, with ASO, siRNA, and small-molecule approaches progressing from early pharmacodynamic studies to large cardiovascular outcomes trials. Available evidence demonstrates substantial reductions in circulating Lp(a) across several investigational approaches, while oral small-molecule inhibitors may offer a potentially more accessible treatment strategy.

However, the relationship between pharmacological Lp(a) lowering and cardiovascular outcome benefit remains unproven. No targeted Lp(a)-lowering therapy has yet demonstrated definitive reductions in cardiovascular events in a completed outcomes trial. Ongoing studies, including Lp(a) HORIZON, OCEAN(a)-Outcomes, ACCLAIM-Lp(a), and MOVE-Lp(a), will determine whether Lp(a) lowering translates into a clinically meaningful cardiovascular risk reduction and define the future role of these therapies in guideline recommendations and clinical practice.

## Figures and Tables

**Table 1:** Characteristics of included clinical trials targeting lipoprotein(a).

NCT Number	Investigational Agent	Therapeutic Class	Phase/Study Status	Number of Participants	Target Population	Trial Focus	Outcome Trial Identification *
NCT06813911	Pelacarsen (with background inclisiran)	ASO	Phase 3/Recruiting	340	Established ASCVD with elevated Lp(a)	Combination pharmacodynamic	—
NCT03070782	Pelacarsen	ASO	Phase 2/Completed	286	Established ASCVD with elevated Lp(a)	Dose-finding	—
NCT06875973	Pelacarsen	ASO	Phase 3/Recruiting	600	Established ASCVD with elevated Lp(a)	Long-term extension	—
NCT06267560	Pelacarsen	ASO	Phase 3/Active, not recruiting	423	Black/Hispanic patients with established ASCVD and elevated Lp(a)	Special population	—
NCT05646381	Pelacarsen	ASO	Phase 2/Recruiting	502	Calcific aortic stenosis with elevated Lp(a)	Aortic stenosis progression	—
NCT04023552	Pelacarsen	ASO	Phase 3/Active, not recruiting	8323	Established ASCVD with elevated Lp(a)	Cardiovascular outcomes	Lp(a) HORIZON CVOT
NCT05900141	Pelacarsen	ASO	Phase 3/Active, not recruiting	41	Elevated Lp(a) (hyperlipoproteinemia[a])	Long-term extension	—
NCT05305664	Pelacarsen	ASO	Phase 3/Completed	51	Elevated Lp(a) (hyperlipoproteinemia[a]) requiring lipoprotein apheresis	Apheresis reduction	—
NCT04270760	Olpasiran (AMG 890)	siRNA	Phase 2/Completed	281	Established ASCVD with elevated Lp(a)	Dose-finding	—
NCT05581303	Olpasiran (AMG 890)	siRNA	Phase 3/Active, not recruiting	7297	Established ASCVD with elevated Lp(a)	Cardiovascular outcomes	OCEAN(a) CVOT
NCT07136012	Olpasiran (AMG 890)	siRNA	Phase 3/Recruiting	11,000	High-risk primary prevention population with elevated Lp(a)	Primary prevention cardiovascular outcomes	CVOT
NCT03626662	Olpasiran (AMG 890)	siRNA	Phase 1/Completed	79	Elevated Lp(a) in adults with cardiovascular risk factors	First-in-human safety/pharmacodynamic	—
NCT07185776	HRS-5632	siRNA	Phase 2/Recruiting	200	Elevated Lp(a) at high cardiovascular risk	Dose-finding and safety	—
NCT06292013	Lepodisiran (LY3819469)	siRNA	Phase 3/Recruiting	16,700	Established ASCVD or high-risk primary prevention with elevated Lp(a)	Cardiovascular outcomes	ACCLAIM-Lp(a) CVOT
NCT04606602	Zerlasiran (SLN360)	siRNA	Phase 1/Completed	70	Elevated Lp(a) in adults without severe comorbidity	First-in-human safety/pharmacodynamic	—
NCT05537571	Zerlasiran (SLN360)	siRNA	Phase 2/Completed	180	Elevated Lp(a) at high risk for ASCVD events	Multiple-dose evaluation	—
NCT07172646	SRSD216	siRNA	Phase 1/2/Recruiting	48	Elevated Lp(a) (hyperlipoproteinemia[a])	Early-phase safety/pharmacodynamic	—
NCT06816264	HRS-5346	Small molecule	Phase 2/Active, not recruiting	120	Elevated Lp(a) at high cardiovascular risk	Dose-finding	—
NCT05563246	Muvalaplin (LY3473329)	Small molecule	Phase 2/Completed	233	Elevated Lp(a) at high cardiovascular risk	Dose-finding	—
NCT07157774	Muvalaplin (LY3473329)	Small molecule	Phase 3/Recruiting	10,450	Established ASCVD with elevated Lp(a)	Cardiovascular outcomes	MOVE-Lp(a) CVOT

* Cardiovascular outcomes trials were defined as studies primarily designed to evaluate major adverse cardiovascular events or other clinical cardiovascular outcomes rather than pharmacodynamic or biomarker-based endpoints.

**Table 2:** Lp(a)-Related Endpoint Assessment Strategies in Included Clinical Trials.

NCT Number	Investigational Agent	Primary Lp(a) Endpoint Format	Additional Lp(a)-Related Assessments	Follow-Up Duration	Dose Level(s)	Dosing Frequency	Route of Administration
NCT06813911	Pelacarsen	Log-transformed concentration change	Threshold achievement analysis, Safety/tolerability monitoring	16 months	80 mg	Once monthly for 12 months	Subcutaneous
NCT03070782	Pelacarsen	Percent change from baseline	Threshold achievement, ApoB/LDL-C, OxPL-apo(a), safety/PK	6 months	20–60 mg	QW, Q2W, or Q4W; maximum 13 doses	Subcutaneous
NCT06875973	Pelacarsen	Absolute concentration change	Safety/tolerability monitoring	48 months	80 mg	Once monthly	Subcutaneous
NCT06267560	Pelacarsen	Log-transformed concentration change	Safety/tolerability monitoring	52 weeks	80 mg	Once monthly	Subcutaneous
NCT05646381	Pelacarsen	Absolute concentration change	Aortic valve progression assessment, Clinical cardiovascular endpoints	36 months	80 mg	Once monthly	Subcutaneous
NCT04023552	Pelacarsen	Time-to-event plus Lp(a) reduction	MACE assessment	4 years	80 mg	Once monthly	Subcutaneous
NCT05900141	Pelacarsen	Composite pharmacodynamic assessment	Long-term safety/tolerability monitoring, Lp(a) durability assessment	60 months	80 mg	Monthly	Subcutaneous
NCT05305664	Pelacarsen	Log-transformed concentration change	Apheresis frequency assessment, ApoB and LDL-C assessment	52 weeks	80 mg	Every 4 weeks (Q4W)	Subcutaneous
NCT04270760	Olpasiran (AMG 890)	Percent change from baseline	ApoB and LDL-C assessment, Pharmacokinetic assessment	48 weeks	10–225 mg	every 12 weeks (Q12W) or 24 weeks (Q24W)	Subcutaneous
NCT05581303	Olpasiran (AMG 890)	Time-to-event plus Lp(a) reduction	MACE assessment	4 years	Not reported	once every 12 weeks (Q12W)	Not reported
NCT07136012	Olpasiran (AMG 890)	Time-to-event plus Lp(a) reduction	MACE assessment, Safety/tolerability monitoring	6.2 years	Not reported	Not reported	Subcutaneous
NCT03626662	Olpasiran (AMG 890)	Composite pharmacodynamic assessment	Safety/tolerability monitoring, Pharmacokinetic assessment	12 months	3–675 mg	Ascending single doses	Subcutaneous
NCT07185776	HRS-5632	Percent change from baseline	Threshold achievement analysis, Safety/tolerability monitoring, Pharmacokinetic assessment	18 months	Not reported	Not reported	Not reported (Injection)
NCT06292013	Lepodisiran (LY3819469)	Time-to-event plus Lp(a) reduction	MACE assessment	4.5 years	Not reported	Not reported	Subcutaneous
NCT04606602	Zerlasiran (SLN360)	Composite pharmacodynamic assessment	Safety/tolerability monitoring, Pharmacokinetic assessment	28 weeks	30–900 mg	Single and multiple dose	Subcutaneous
NCT05537571	Zerlasiran (SLN360)	Time-averaged percent reduction	ApoB and LDL-C assessment	60 weeks	300 mg, 450 mg	Q16W-Q24W (2–3 doses)	Subcutaneous
NCT07172646	SRSD216	Composite pharmacodynamic assessment	Safety/tolerability monitoring, Pharmacokinetic assessment	52 weeks	Not reported	Single and multiple dose	Subcutaneous
NCT06816264	HRS-5346	Percent change from baseline	Threshold achievement analysis, Safety/tolerability monitoring, Pharmacokinetic assessment	16 weeks	Not reported	Not reported	Oral
NCT05563246	Muvalaplin (LY3473329)	Percent change from baseline	Threshold achievement analysis, ApoB and LDL-C assessment, Biomarker assessment, Pharmacokinetic assessment	12 weeks	10 mg, 60 mg, 240 mg	Once daily for 12 weeks	Oral
NCT07157774	Muvalaplin (LY3473329)	Time-to-event plus Lp(a) reduction	MACE assessment	5.25 years	Not reported	Not reported	Oral

MACE = major adverse cardiovascular event; PK = pharmacokinetic; OxPL-apo(a) = oxidized phospholipids on apolipoprotein(a).

**Table 3:** Summary of Published Efficacy and Durability Characteristics of Investigational Lipoprotein(a)-Lowering Therapies.

Investigational Agent	Therapeutic Class	Representative Published Trial	Maximum Reported Lp(a) Reduction	Durability of Effect	Clinical Development Status	Key Clinical Notes
Pelacarsen	ASO	Phase 2 AKCEA-APO(a)-LRx trial; Lp(a)HORIZON	~80%	Sustained during chronic dosing	Phase 3 outcomes ongoing	Most clinically advanced ASO therapy; monthly administration
Olpasiran	siRNA	OCEAN(a)-DOSE	~95–100%	Persistent >1 year after last dose	Phase 3 outcomes ongoing	Long-acting siRNA supporting infrequent dosing intervals
Lepodisiran	siRNA	Phase 1 first-in-human trial	~95–97%	Sustained ~94% reduction for ~11 months after single dose	Phase 3 outcomes ongoing	Extended-duration single-dose suppression profile
Zerlasiran (SLN360)	siRNA	Phase 1 NCT04606602; ALPACAR-360	~98–99%	Sustained ~90% reduction for ~6 months after high-dose administration	Phase 2 clinical development	Supports quarterly or semiannual dosing strategies
Muvalaplin	Small-molecule inhibitor	Nicholls et al. Phase 2 Trial	~86%	Sustained during daily oral administration	Phase 2 clinical development	First oral Lp(a)-targeting therapy in clinical development

Data summarized from representative peer-reviewed clinical trials and published efficacy reports of investigational lipoprotein(a)-lowering therapies. Reported efficacy values represent approximate maximal reductions in circulating lipoprotein(a) observed under specific study conditions and are intended for descriptive purposes only. These values should not be interpreted as direct comparative estimates between therapies. Differences in study populations, baseline Lp(a) concentrations, dosing regimens, follow-up duration, endpoint definitions, and assay methodologies limit cross-trial comparisons.

## Data Availability

No new datasets were generated. All data analyzed in this study were obtained from the publicly accessible ClinicalTrials.gov registry.

## Supplementary Materials

The following supporting information can be downloaded at: https://www.mdpi.com/2077-0383/15/13/5233/s1. Supplementary Table S1: Key Design and Treatment Characteristics of Included Lp(a)-Targeted Clinical Trials; Supplementary Table S2: Summary of Reported Safety and Tolerability Findings of Investigational Lp(a)-Lowering Therapies.

## Abbreviations

The following abbreviations are used in this manuscript:

Apo(a)	Apolipoprotein(a)
ApoB	Apolipoprotein B
ASCVD	Atherosclerotic Cardiovascular Disease
ASO	Antisense Oligonucleotide
CVD	Cardiovascular Disease
CVOT	Cardiovascular Outcome Trial
LDL-C	Low-Density Lipoprotein Cholesterol
Lp(a)	Lipoprotein(a)
MACE	Major Adverse Cardiovascular Event
OxPL-apo(a)	Oxidized Phospholipids on Apolipoprotein(a)
PCSK9	Proprotein Convertase Subtilisin/Kexin Type 9
PK	Pharmacokinetic
siRNA	Small Interfering RNA
