# Supplementary material for: Emerging Therapies Targeting Lipoprotein(a): A Clinical Trial Landscape Review of Investigational Lp(a)-Lowering Therapies
Source: J Clin Med. 2026 Jul 4;15(13):5233. doi: 10.3390/jcm15135233 (PMC13363407; doi:10.3390/jcm15135233)
Supplement: Supplementary file 1 [file jcm-15-05233-s001.zip › jcm-4385066-supplementary.pdf]

Supplementary Table S1. Key Design and Treatment Characteristics of Included Lp(a)-Targeted Clinical Trials

NCT Number	Study Title	Investigational Agent	Allocation	Masking	Comparator	Route of Administration	Dose Level(s)	Dosing Frequency
NCT06813911	Lp(a) Lowering Study of Pelacarsen (TQJ230) With Background Inclisiran in Participants With Elevated Lp(a) and Established ASCVD	Pelacarsen	Randomized	Double-blind	Placebo	Subcutaneous	80 mg	Once monthly for 12M
NCT03070782	Phase 2 Study of ISIS 681257 (AKCEA-APO(a)-LRx) in Participants With Hyperlipoproteinemia(a) and Cardiovascular Disease	Pelacarsen	Randomized	Triple-blind	Placebo	Subcutaneous	20 mg Q4W, 20 mg Q2W, 20 mg QW, 40 mg Q4W, 60 mg Q4W	Q4W, Q2W, QW for a maximum of 13 doses
NCT06875973	Pelacarsen Roll-over Extension Program	Pelacarsen	Non-randomized	Open-label	None	Subcutaneous	80 mg	Once monthly
NCT06267560	Lp(a) Lowering Study of Pelacarsen (TQJ230) in US Black/African American and Hispanic Participants With Elevated Lp(a) and Established ASCVD	Pelacarsen	Randomized	Quadruple-blind	Placebo	Subcutaneous	80 mg	Once monthly
NCT05646381	A Multicenter Trial Assessing the Impact of Lipoprotein(a) Lowering With Pelacarsen (TQJ230) on the Progression of Calcific Aortic Valve Stenosis	Pelacarsen	Randomized	Double-blind	Placebo	Subcutaneous	80 mg	Once monthly
NCT04023552	Assessing the Impact of Lipoprotein (a) Lowering With Pelacarsen (TQJ230) on Major Cardiovascular Events in Patients With CVD (Lp(a)HORIZON)	Pelacarsen	Randomized	Double-blind	Placebo	Subcutaneous	80 mg	Once monthly
NCT05900141	An Open Label Extension (OLE) Study to Evaluate Long-term Safety and Tolerability of Pelacarsen (TQJ230) (OLE)	Pelacarsen	Non-randomized	Open-label	None	Subcutaneous	80 mg	Monthly
NCT05305664	A Multicenter Trial Assessing the Impact of Lipoprotein(a) Lowering With Pelacarsen (TQJ230) on the Rate of Weekly Lipoprotein Apheresis Sessions in Patients With	Pelacarsen	Randomized	Quadruple-blind	Placebo	Subcutaneous	80 mg	Every 4 weeks (Q4W)

	Hyperlipoproteinemia(a) and Established Cardiovascular Disease in Germany							
NCT04270760	Olpasiran Trials of Cardiovascular Events And LipoproteiN(a) Reduction - DOSE Finding Study	Olpasiran (AMG 890)	Randomized	Double-blind	Placebo	Subcutaneous	10 mg Q12W, 75 mg Q12W, 225 mg Q12W, 225 mg Q24W	every 12 weeks (Q12W) or 24 weeks (Q24W)
NCT05581303	Olpasiran Trials of Cardiovascular Events and Lipoprotein(a) Reduction (OCEAN(a)) - Outcomes Trial	Olpasiran (AMG 890)	Randomized*	Double-blind*	Placebo	Not reported	Not reported	once every 12 weeks (Q12W)
NCT07136012	OCEAN(a)-PreEvent - Olpasiran Trials of Cardiovascular Events And LipoproteiN(a) Reduction to Prevent First Major Cardiovascular Events	Olpasiran (AMG 890)	Randomized	Double-blind	Placebo	Subcutaneous	Not reported	Not reported
NCT03626662	Safety, Tolerability, Pharmacokinetics and Pharmacodynamics Study of AMG 890 in Subjects With Elevated Plasma Lipoprotein(a)	Olpasiran (AMG 890)	Randomized	Triple-blind	Placebo	Subcutaneous	3-675 mg	Ascending Single doses
NCT07185776	A Study of HRS-5632 in Adult Participants With Elevated Lipoprotein(a) at High Risk for Cardiovascular Events	HRS-5632	Randomized	Quadruple-blind	Placebo	Injection	Not reported	Not reported
NCT06292013	A Study to Investigate the Effect of Lepodisiran on the Reduction of Major Adverse Cardiovascular Events in Adults With Elevated Lipoprotein(a) - ACCLAIM-Lp(a)	Lepodisiran (LY3819469)	Randomized	Double-blind	Placebo	Subcutaneous	Not reported	Not reported
NCT04606602	Study to Investigate Safety, Tolerability, PK and PD Response of SLN360 in Subjects With Elevated Lipoprotein(a)	Zerlasiran (SLN360)	Randomized	Double-blind	Placebo	Subcutaneous	30 mg, 100 mg, 200 mg, 300 mg, 600 mg, 900 mg	Single and multiple dose
NCT05537571	Evaluate SLN360 in Participants With Elevated	Zerlasiran (SLN360)	Randomized	Double-blind	Placebo	Subcutaneous	300 mg Q16W,	multiple doses (2-3)

	Lipoprotein(a) at High Risk of Atherosclerotic Cardiovascular Disease Events						300 mg Q24W, 450 mg Q24W	
NCT07172646	A Study of SRSD216 in Patients With Elevated Lipoprotein (a)	SRSD216	Randomized	Triple-blind	Placebo	Subcutaneous	Not reported	Single and multiple dose
NCT06816264	A Study of HRS-5346 in Adult Participants With Elevated Lipoprotein(a) at High Risk for Cardiovascular Events	HRS-5346	Randomized	Quadruple-blind	Placebo	Oral	Not reported	Not reported
NCT05563246	A Study of LY3473329 in Adult Participants With Elevated Lipoprotein(a) at High Risk for Cardiovascular Events	Muvalaplin (LY3473329)	Randomized	Double-blind	Placebo	Oral	10 mg, 60 mg, 240 mg	Once daily for 12 weeks
NCT07157774	Assessing the Impact of Muvalaplin on Major Cardiovascular Events in Adults With Elevated Lipoprotein(a) (MOVE-Lp(a))	Muvalaplin (LY3473329)	Randomized	Double-blind	Placebo	Oral	Not reported	Not reported

Supplementary Table S2. Summary of Reported Safety and Tolerability Findings of Investigational Lp(a)-Lowering Therapies

Therapeutic Agent	Adverse events	Important Laboratory Findings	Overall Safety and Tolerability	Key References
Pelacarsen	Injection-site reactions (erythema, pain, pruritus); mild influenza-like symptoms; nasopharyngitis; headache.	Generally small, reversible changes in liver enzymes; no consistent clinically relevant changes in creatine kinase or hematologic parameters.	Generally well tolerated in phase 1–2 studies. Injection-site reactions were the most common adverse events. No major safety signal was identified, and serious adverse events were infrequent and typically unrelated to treatment. Long-term safety remains under evaluation in ongoing phase 3 trials.	[13, 15]
Olpasiran	Mild injection-site reactions (pain, erythema, swelling); headache; nasopharyngitis; upper respiratory tract infections.	No consistent clinically meaningful abnormalities in liver enzymes, renal function, or platelet counts reported in phase 1–2 trials.	Generally well tolerated across evaluated doses. Adverse events were predominantly mild to moderate, most commonly injection-site reactions. No major drug-related safety signals were identified, and serious adverse events were uncommon. Longer-term safety and rare events will be defined by ongoing phase 3 outcomes trials.	[12, 16]
Lepodisiran	Injection-site reactions (transient pain, erythema); headache; fatigue; mild gastrointestinal symptoms in some participants.	Transient, dose-related increases in liver enzymes and C-reactive protein were reported in some participants; no consistent dose-limiting laboratory safety signal was identified.	Early-phase studies demonstrated favorable tolerability with predominantly mild adverse events. No major safety signal was identified, although safety data remain limited by relatively small sample sizes and follow-up durations.	[14]
Zerlasiran (SLN360)	Injection-site reactions (tenderness, erythema,	Transient, dose-related increases in liver enzymes and C-reactive protein reported in some	Available phase 1–2 data suggest acceptable tolerability with infrequent dosing schedules. No consistent serious safety signals have been reported, although longer-term safety remains to be established.	[22]

	induration); headache; myalgia; mild flu-like symptoms.	participants; generally resolved without sequelae.		
Muvalaplin	Mild gastrointestinal symptoms (nausea, diarrhea, dyspepsia); headache; nasopharyngitis; dizziness.	No consistent pattern of clinically significant liver enzyme elevations or other laboratory toxicities in phase 1–2 datasets; routine chemistry and hematology largely stable.	Available phase 1–2 data indicate good tolerability of daily oral administration, with predominantly mild adverse events. No major safety signals have been identified to date. Long-term safety and potential off-target effects require confirmation in the ongoing MOVE-Lp(a) outcomes program.	[11]

Abbreviations: Lp(a), lipoprotein(a). Safety findings are summarized from published clinical studies available at the time of review. Direct comparisons between therapies should be interpreted cautiously because studies differed substantially in design, sample size, follow-up duration, adverse-event reporting methods, and developmental phase.
